# Multivariate analysis of grain amaranth (*Amaranthus* spp.) accessions to quantify phenotypic diversity

**DOI:** 10.1016/j.heliyon.2022.e11613

**Published:** 2022-11-15

**Authors:** Saujan Bashyal, Ashmita Upadhyay, Dipendra Kumar Ayer, Prabesh Dhakal, Bimochana G.C., Jiban Shrestha

**Affiliations:** a Department of Agronomy, Plant Breeding and Agricultural Statistics, Institute of Agriculture and Animal Science, Tribhuvan University, Lamjung Campus, Sundarbazar, Lamjung, Nepal; b Nepal Agricultural Research Council, National Plant Breeding and Genetics Research Centre, Khumaltar, Lalitpur, Nepal

**Keywords:** *Amaranthus*, Cluster analysis, Diversity, Mahalanobis distance, PCA

## Abstract

Twelve amaranth accessions were evaluated in a randomized complete block design with three replications in the research field of the Institute of Agriculture and Animal Science, Lamjung, Sundarbazar, Lamjung, Nepal from March to July 2021 to assess the phenotypic diversity. Principal Component Analysis (PCA) showed that only two principal components were significant with their eigenvalues >1 and combinedly accounted for 88.3% of the total variance. PC1, which explained 71.9% of the variance, was highly and positively contributed by days to 50% inflorescence, plant height, leaf length, leaf width, petiole length, 1000 seed weight, and grain yield. PC1 was negatively affected by the number of primary branches per plant and the number of leaves per plant. PC2, which explained 16.4% of the variance, distinguished plants with high number of leaves and a higher inflorescence length. The accessions were grouped into 4 clusters. Cluster 2 had the greatest intracluster distance (D^2^ = 13.19), while Cluster 3 and 4 had the greatest intercluster distance (D^2^ = 21.73), followed by Cluster 2 and 3 (D^2^ = 17.37). Cluster 1 had the highest number of leaves per plant and the lowest yield. Cluster 2 had the maximum grain yield and plant height. Cluster 3 had the lowest inflorescence length, and cluster 4 had the highest leaf length and 1000 seed weight.

## 1 Introduction

The *Amaranthaceae* family includes the genus *Amaranthus*, which has over 75 species throughout the world. This big genus contains a distinct group of 10 dioecious species that have separate male and female plants (Steckel, 2007). Amaranth belongs to *Amaranthaceae* family, Caryophyllales order, and *Amaranthus* genus (Wolosik and Markowska, 2019). The majority of these nutrient-rich pseudocereal crops are grown in temperate and tropical areas for use as a grain or leafyvegetable. According to Dincă et al. (2018), it has leaves that are rich in oxalates, calcium, folate, polyphenols, saponins, tannins, and vitamins (A and C). Since it is the least expensive and easiest to obtain source of minerals, vitamins, and protein among leafy greens, it is also known as the "poor man's vegetable" (Varalakshmi, 2004). Minerals (105 % Daily Value), fibers, minerals (40% DV), iron (29 % DV), and selenium (20 % DV) are all said to be present in nutrient-rich grains (Alegbejo, 2014). More protein is present in this crop than other cereal crops, and the protein is of higher quality since it includes more lysine than cereals like rice, wheat, and maize do (Alegbejo, 2014). Amino acid balance in amaranth protein is closer to the optimal equilibrium required in human nutrition (Bhattarai, 2018). Amaranths are easy-to-cook grains that have the potential to improve protein or amino acid deficits, increase mineral content (Fe, Zn), and supplement vegetarian diets with protein (Thapa and Blair, 2018). The morphological variability of grain amaranth is extensive, and it is tolerant of a wide range of ecological and geographic conditions (Gelotar et al., 2019). *A. caudatus* L. and *A. hypochondriacus* L. are the two main species of grain amaranth farmed in Nepal; the former is only grown in hilly places, while the latter is widely grown in places above 3000 m. *A. cruentus* is also found in various regions of the nation (Jha et al., 1991). Only three promising varieties—RatoMarse, LadiMarse, and Suntale latte—have been made available in Nepal, with RatoMarse and LadiMarse being recommended for high hills and mountains and Suntale latte for mid-to high hills (Joshi et al., 2016). The Himalayan region's traditional crop, grain amaranth (*A. hypochondriacus* L.), is grown there as a mixed crop for sustenance in areas with poor farming practices and relatively little rainfall (Kandel et al., 2021). Jha et al. (1991) reported that the grain yield ranged from 380 to 1480 kg/ha.

Multivariate analytical techniques are frequently used in the study of genetic diversity. Cluster Analysis and Principal Component Analysis (PCA) are presently the most often used methodologies. They seem to be particularly well adapted to evaluating yield-related parameters (Melchinger, 1993; Johns et al., 1997; Brown-Guedira et al., 2000). With the use of cluster analysis, variations can be clustered into homogeneous and heterogeneous groups, guaranteeing that variety within a clusterper form similarly across locations (Kandel et al., 2018). Many researchers have used principal component analysis to investigate genetic variation among maize genotypes because it yields a small number of components that account for the majority of data changes (Asare, 2016).

The objective of this study was to assess the variability among amaranthus accessions using cluster analysis and principal component analysis.

## 2 Materials and methods

### 2.1 Experimental site

The study was carried out at the research field of the Institute of Agriculture and Animal Science, Lamjung, Sundarbazar, Lamjung, Nepal from March to July 2021. The study site is located at 28.12°N latitude,84.41°E longitude, and 620 m elevation. The experimental area's soil type was sandy loam.

### 2.2 Plant materials

Twelve Amaranthus accessions were collected from the National Agriculture Genetic Resource Center (NAGRC), Khumaltar, Lalitpur, Nepal. The name list of the accessions is given in Table 1.

**Table 1:** List of 12 accessions of amaranth collected from NAGRC, Khumaltar, Lalitpur, Nepal.

Source of collection	Accessions
National Agriculture Genetic Resource Center (NAGRC), Khumaltar, Lalitpur, Nepal.	CO-110, CO-1239, CO-2435, CO-6958, CO-1241, CO-3616, CO-1888, CO-984, NGR–CO–9677, CO-7790, CO-1890, CO- 1242

### 2.3 Experimental design, treatment details and cultural practices

The experiment was laid out in a randomized complete block design with three replications. To ensure the uniformity in sowing, seeds were mixed with fine sand. Initially, line sowing was done, maintaining 40 cm spacing between the lines. Gradual thinning was carried out until the inter-plant distance of 40 cm was achieved. Standard agronomic practices were followed.

### 2.4 Data collection

Data was recorded for eleven quantitative traits at different stages as suggested by the International Union for the Protection of New Varieties of Plants (UPOV) descriptor. A detailed description of quantitative traits and their evaluation phase is presented in Table 2.

**Table 2:** Quantitative traits of amaranth with their evaluation phase.

Quantitative character	Description	Evaluation stage
Days to 4 leaf stage	Days from sowing to 4 leaf stage	At four-leaf stage
Days to flowering	Days from sowing to 50% flowering	At flowering stage
Plant height	Height of plant from base to the tip of inflorescence	At maturity
Leaf length	From base of leaf to tip in 8^th^ leaf	At maturity
Leaf width	In the broadest part of 8^th^ leaf	At maturity
Number of leaves per plant	Total number of leaves in the sample plant	At maturity
Number of branches per plant	Total number of primary branches per plant in the sample plant	At maturity
Petiole length	Length of petiole of the 8^th^ leaf	At maturity
Inflorescence length	Length of terminal inflorescence from base of inflorescence to its tip	At maturity
1000 seed weight	Weight of 1000 randomly selected seeds of each plot	After harvest
Seed yield per ha	Plot yield converted into yield per ha.	After harvest

### 2.5 Statistical analysis

Data entry was done in MS-EXCEL 2016. R Studio V4.1.1 was used for statistical analysis. Principal Component Analysis was performed using the FactoMineR package and eigenvalues, eigenvectors, and 2D-biplots were obtained using the factoextra package. Cluster analysis was performed by Tocher's method using squared generalized Mahalanobis distance (D^2^) as suggested by da Silva and Dias (2013).

## 3 Results and discussion

### 3.1 Principal component analysis

**Figure 1:**
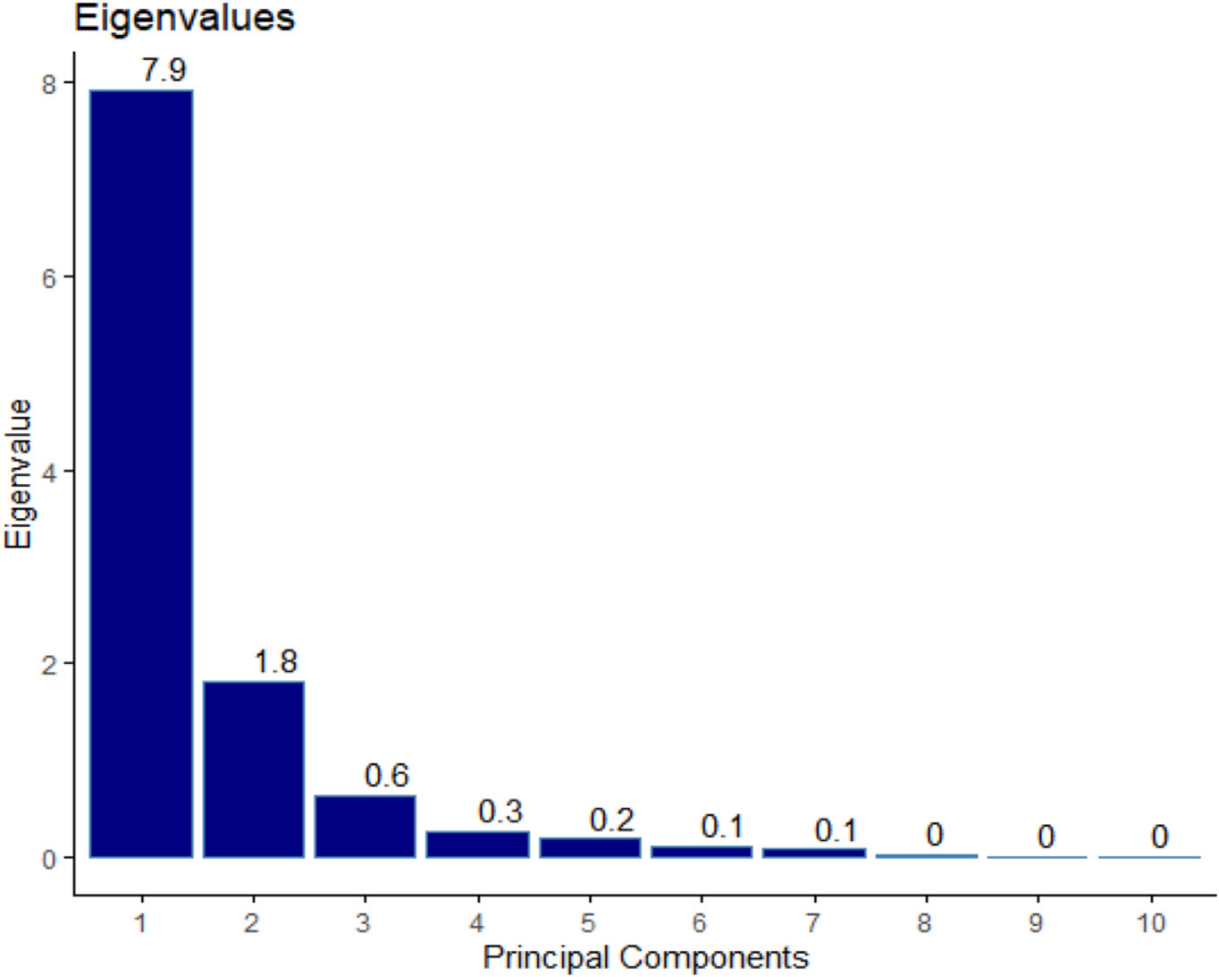
Eigenvalue of principal components of *Amaranthus* spp. accessions.

**Figure 2:**
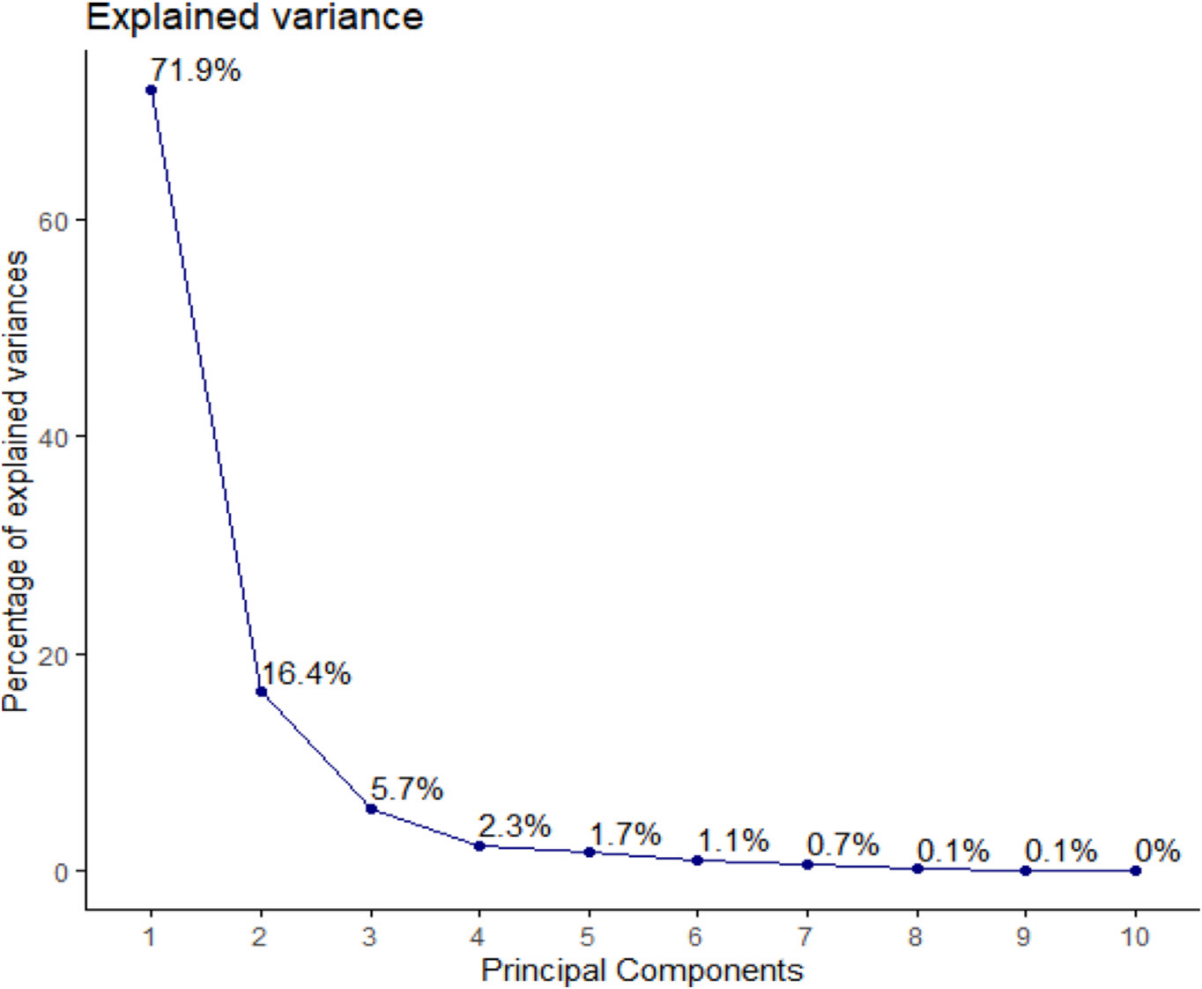
Contribution of each principal component to total explained variance in the phenotypic diversity of *Amaranthus* spp.

**Figure 3:**
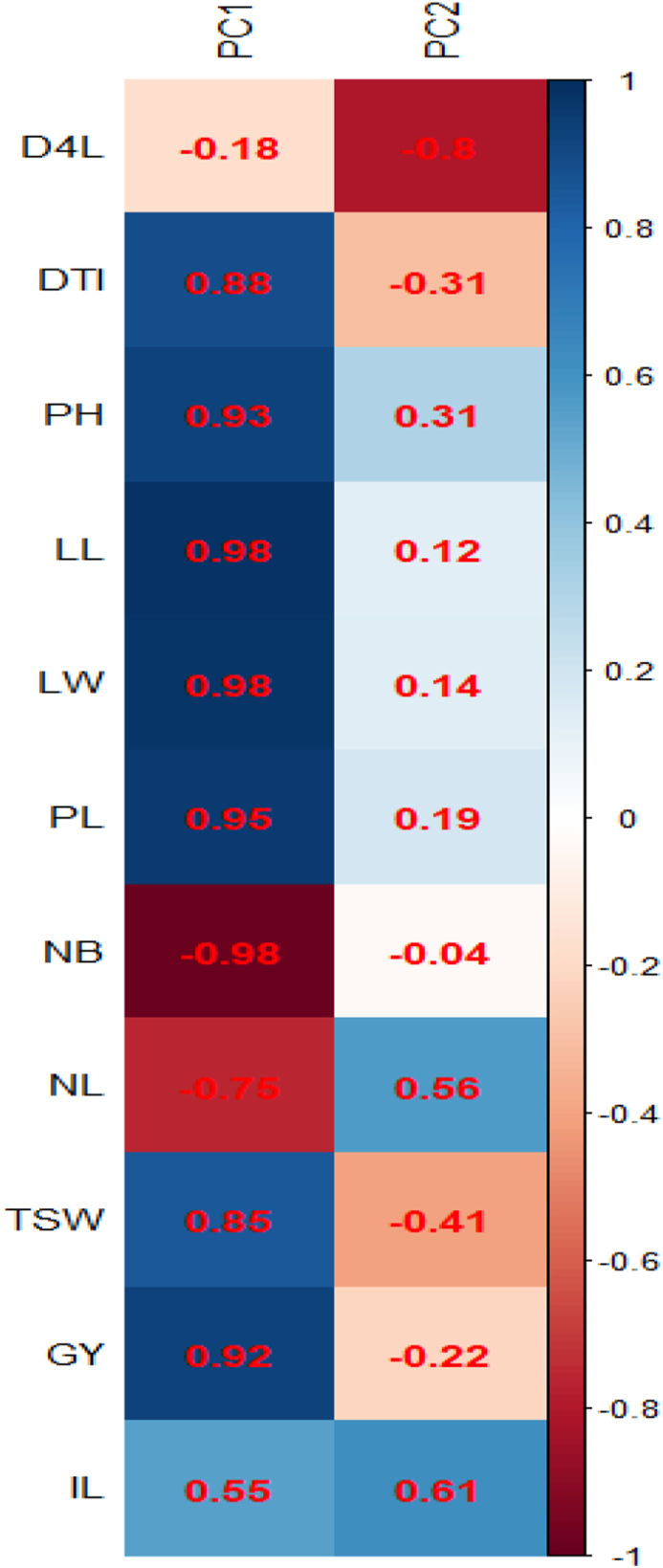
Vector loading and its eigenvalue for quantitative traits of 12 accessions of *Amaranthus* spp*.* The blue color indicates the positive contribution, and red color indicates the negative contribution. (D4L: Days to 4 leaf stage, DTI: Days to 50% Inflorescence, PH: Plant height, LL: Leaf Length, LW: Leaf Width, PL: Petiole length, NB: Number of branches per plant, NL: Number of leaves per plant, TSW: 1000 seed weight, GY: Grain Yield per ha, IL: Inflorescence length).

**Figure 4:**
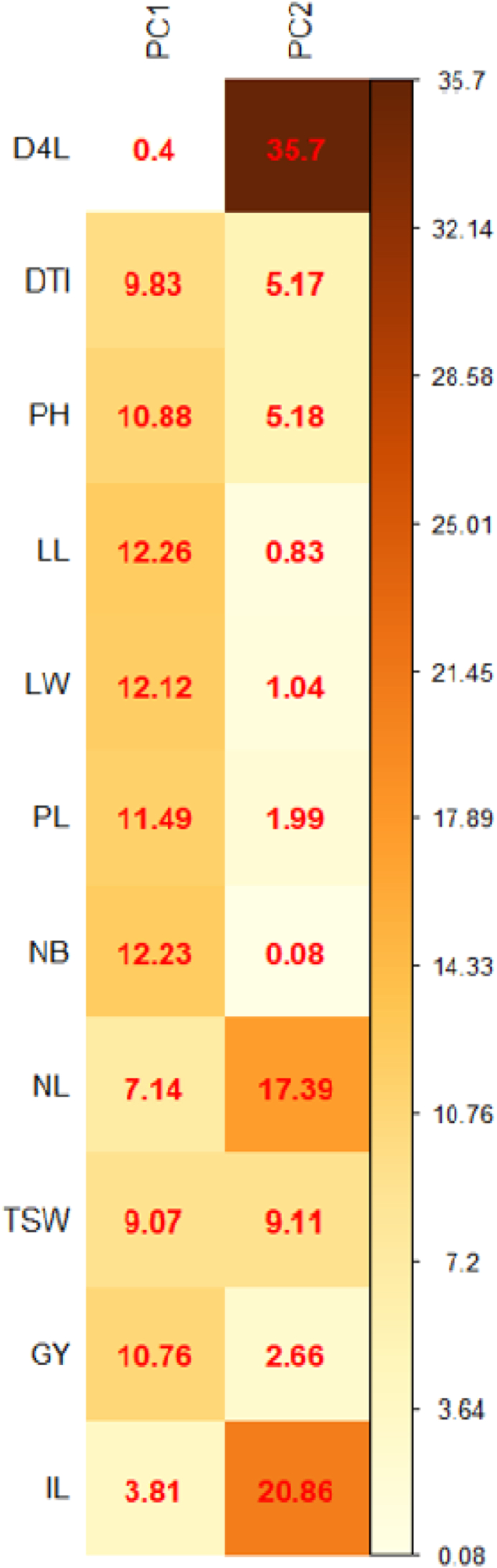
Percentage contribution of 11 quantitative traits on PC1 and PC2. (D4L: Days to 4 leaf stage, DTI: Days to 50% Inflorescence, PH: Plant height, LL: Leaf Length, LW: Leaf Width, PL: Petiole length, NB: Number of branches per plant, NL: Number of leaves per plant, TSW: 1000 seed weight, GY: Grain Yield per ha, IL: Inflorescence length).

The PCA revealed the first two most informative principal components with eigenvalues of 7.9 and 1.8, respectively (Figure 1). The PCA findings demonstrated that PC1 alone explained 71.9 % variance while PC2 explained 16.4 % (Figure 2). They accounted for 88.3% of the overall variance when taken together. Only PC1 and PC2 were chosen for additional statistical analysis since both of their eigenvalues were greater than 1. PC1 was positively influenced by the days to 50% inflorescence, plant height, leaf length, leaf breadth, petiole length, inflorescence length, 1000 seed weight, and grain yield. Accessions NGR-CO 9677, CO-984, and CO-1242 performed best in these traits. The days to the fourth leaf stage, the quantity of primary branches per plant, and the number of leaves per plant affected PC1 negatively. Figure 3 shows that yield and yield-attributing traits were distinct parameters in PC1. Akin-Idowu et al. (2016)obtained similar results. These traits had negative correlation with the number of leaves and branches per plant which had positively contributed to PC2. Sarker et al. (2018)also had similar results. PC2 was negatively affected by days to 4 leaves, days to 50% inflorescence, 1000 seed weight, and grain yield. In contrast, PC2 showed a strong positive correlation with the number of leaves and inflorescence length. Although the inflorescence length contributed positively in PC1, it had a greater positive contribution in PC2. The most significant contributors to PC1, accounting for 71.9 % variance, were leaf length (12.26%), number of branches per plant (12.23%), and leaf width (12.12%) (Gerrano et al., 2015) also identified leaf aera and leaf area index as the main contributors to the variation. Figure 4 shows that PC2 was similarly influenced by days to 4 leaf stages (35.7%), number of leaves per plant (17.39%), inflorescence length (20.86%), and 1000 seed weight (9.11%).

**Figure 5:**
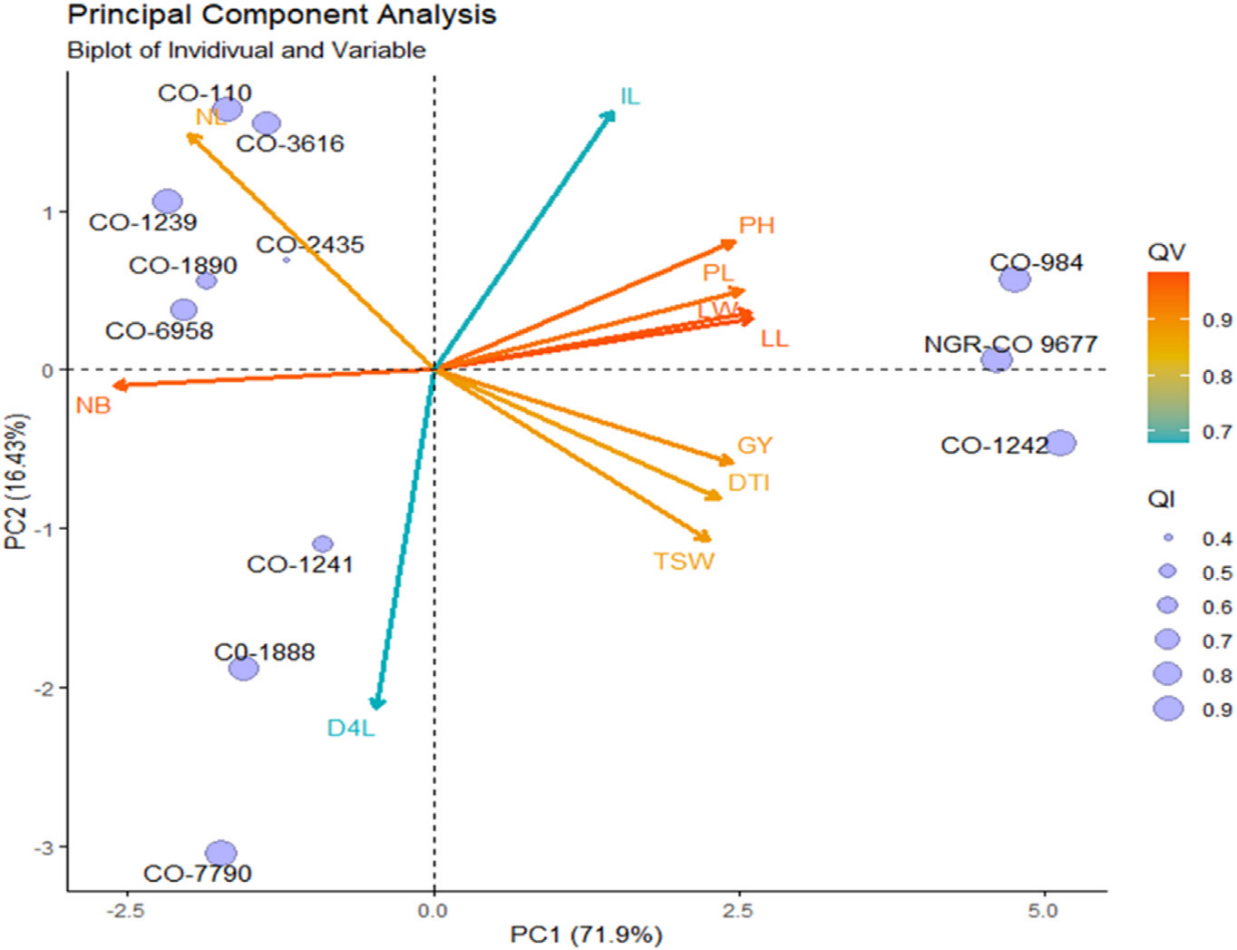
The biplot for 12 *Amaranthus* spp. accessions for PC1 and PC2. The arrows show the contribution (magnitude and direction) of the trait in PC1 and PC2. QI is the quality of representation of individuals, and QV is the quality of representation of variables (D4L: Days to 4 leaf stage, DTI: Days to 50% Inflorescence, PH: Plant height, LL: Leaf Length, LW: Leaf Width, PL: Petiole length, NB: Number of branches per plant, NL: Number of leaves per plant, TSW: 1000 seed weight, GY: Grain Yield per ha, IL: Inflorescence length).

The contribution of each trait in PC1 and PC2 is given in Figure 5. The accessions were grouped into various clusters using PCA. Three accessions (CO-984, NGR-C0 9677, and CO-1242) were grouped in PC1 had higher yield, plant height, leaf length, leaf breadth, and petiole length. Similarly, PC2 identified low-yielding accessions as having more leaves and branches per plant. The most significant contributors to variance were plant height, petiole length, leaf length, leaf breadth, and number of branches, followed by number of leaves per plant, grain yield, days to inflorescence, and 1000 seed weight.

### 3.2 Cluster analysis

Twelve Amaranth accessions were compared using a pairwise squared generalized Mahalanobis distance (D^2^) to determine how genetically distinct they were from one another. As indicated by da Silva and Dias (2013), clustering was carried out using Tocher's approach utilizing this distance. The four clusters among accessions were formed and is given in Table 3. With eight accessions, Cluster 1 was the largest cluster; Clusters 2 and 3 each had two accessions; while Cluster 4 only had one accession. Table 4 displays the intracluster and intercluster distances. The highest intracluster distance was found in cluster 2 (D^2^ = 13.19), followed by cluster 1 (D^2^ = 11.56). Cluster 3 and cluster 4 had 0 intracluster distances because of single accessions in those clusters. The maximum intercluster distance (D^2^ = 21.43) was found between cluster 3 and cluster 4, followed by cluster 2 and cluster 3 (D^2^ = 17.36), which showed high genetic divergence between them. Table 5 shows the mean performance of each cluster on different characters, and a wide range of mean values were seen in different clusters. Cluster 2 had the highest grain yield (1264 kg/ha), followed by cluster 4 (1169 kg/ha), cluster 3 (862 kg/ha) and cluster 1, with the least yield (812 kg/ha). Cluster 2 was distinguished for the highest value of plant height (168cm), leaf width (11 cm), days to inflorescence (62 days) and petiole length (13 cm) (Oboh, 2007). found that accessions in a cluster with low values for plant height and leaf length also had lower values for seed characters. The results of Ahammed et al. (2013) and Dhakal et al. (2020) revealed that distinct clusters of genotypes obtained from the same district. Aiken-Idowu et al. (2016) noted that accessions from the same geographic area were grouped together, which may indicate that some features are more specific to certain geographical places than others. In general, the more genetic heterosis there is, the more members of genetically divergent clusters are projected to have high diversity and the potential to create transgressive segregations with a lot of heterotic consequences (Chandra et al., 2007) (Venkatesh et al., 2014). As a result, by crossing accessions of far-off and effective clusters, superior hybrids could be created.

**Table 3:** Grouping of 12 amaranth accessions into 4 clusters.

Cluster	Number of accessions	Name of accessions
1	8	CO-1241, C0-1888, CO-6958, CO-2435, CO-3616, CO-1239, CO-110 and CO-1890
2	2	CO-1242 and CO-984
3	1	CO-7790
4	1	NGR-CO 9677

**Table 4:** Intracluster (bold and diagonal) and intercluster distances between the clusters.

	Cluster 1	Cluster 2	Cluster 3	Cluster 4
Cluster 1	**11.56**			
Cluster 2	16.27	**13.19**		
Cluster 3	14.26	17.37	**0.00**	
Cluster 4	16.94	17.02	21.73	**0.00**

**Table 5:** Mean performance of traits in clusters.

Clusters	D4L (days)	DTI (days)	PH (cm)	LL (cm)	LW (cm)	PL (cm)	NB	NL	TSW (g)	GY (kg/ha)	IL (cm)
1	18.33	47.83	97.21	10.12	4.94	6.06	4.96	141.00	0.58	812.95	49.15
2	18.33	62.67	168.76	18.91	11.06	13.29	0.00	50.20	0.69	1264.48	54.76
3	19.33	49.33	75.98	9.00	4.33	5.07	5.00	79.00	0.66	862.71	40.40
4	18.00	61.67	154.84	19.33	9.88	12.04	0.00	36.33	0.73	1169.67	53.85

D4L: Days to 4 leaf stage, DTI: Days to 50% Inflorescence, PH: Plant height, LL: Leaf Length, PL: Petiole length, NB: Number of branches per plant, NL: Number of leaves per plant, TSW: 1000 seed weight, GY: Grain Yield, IL: inflorescence length.

From Table 6 grain yield (GY) and plant height (PH) were found highest in CO-984 accession, leaf length and thousand seed weight (TSW) were highest in NGR-CO 9 accession, inflorescence length (IL) was highest in CO-1242, whereas, number of branches per plant (NB) was highest in CO-1241 and CO-3616 accession and number of leaves per plant (NL) was highest in CO-110 accession. Coefficient of variation and standard error of mean were calculated as shown in Table 6.

**Table 6:** Agro-morphological trait of different *Amaranthus* spp. accessions.

Accessions	D4L	DTI	PH	LL	LW	PL	NB	NL	TSW	GY	IL
CO-110	18	45	112.23	11.42	5.58	6.67	5	199.66	0.58	729.88	49.26
CO-2435	18.67	45.33	99.28	10.14	4.93	8.74	4.33	136	0.58	727.58	55.45
CO-1242	18.67	62.33	167.14	19.05	10.68	13.08	0	51	0.72	1345.38	56.53
CO-1890	18.67	46.67	90.39	9.57	4.77	5.41	4.67	160.33	0.53	909.17	53.69
CO-7790	19.33	49.33	75.98	9	4.33	5.07	5	79	0.66	862.71	40.4
CO-6958	18	43.67	85.46	8.86	4.3	5.23	5	118.67	0.58	831.42	47.06
CO-1239	18	43.67	89.82	9.42	4.75	5.12	5	136.67	0.53	796.88	49.62
CO-3616	18	47.33	125.17	11.45	5.77	6.94	5.33	182.33	0.58	724.96	49.41
CO-984	18	63	170.37	18.77	11.43	13.5	0	49.4	0.67	1183.58	53
NGR-CO 9677	18	61.67	154.84	19.33	9.88	12.04	0	36.33	0.73	1169.67	53.85
CO-1241	18.67	58.33	90.36	10.43	4.76	5.57	5.33	101	0.61	897.71	48.6
C0-1888	18.67	52.67	84.92	9.66	4.66	4.8	5	93.33	0.62	886.04	40.11
Mean	18.39	51.58	112.16	12.26	6.32	7.68	3.72	111.98	0.62	922.08	49.75
SEM	0.129	2.22	9.84	1.21	0.772	0.961	0.652	15.4	0.019	53.8	1.53
CV%	4.33	12.5	12.75	11.09	12.31	25.05	13.37	42.11	10	21.05	11.67
F test	∗	∗∗	∗∗	∗∗	∗∗	∗∗	∗∗	∗∗	∗∗	∗∗	∗∗

D4L: Days to 4 leaf stage, DTI: Days to 50% Inflorescence, PH: Plant height, LL: Leaf Length, PL: Petiole length, NB: Number of branches per plant, NL: Number of leaves per plant, TSW: 1000 seed weight, GY: Grain Yield, IL: Inflorescence length, ∗ = Significant at 0.05 level of significance, ∗∗ = Significant at 0.01 level of significance.

## 4 Conclusion

The multivariate statistical tools (PCA and cluster analysis) revealed that the *Amaranthus* spp*.* accessions had a wide range of phenotypic diversity. The accessions CO-1242 and CO-984 produced the highest grain yield of 1345.38 kg/ha and 1183.58 kg/ha respectively. PC1 was highly and positively contributed by leaf length, leaf width, petiole length, plant height, 1000 seed weight, and grain yield, and a high correlation was observed between these traits. Hence, they should be considered in breeding programs. Cluster analysis identified four clusters, each having different performances, and significant genetic divergence between clusters was seen. The highest inter-cluster distance was exhibited by Cluster 3 and 4, followed by 2 and 3.

## Declarations

### Author contribution statement

Saujan Bashyal, Ashmita Upadhyay, Prabesh Dhakaland Bimochana G.C.: Conceived and designed the experiments; Performed the experiments; Analyzed and interpreted the data; Wrote the paper.

Dipendra Kumar Ayer and Jiban Shrestha: Reviewed initial draft of manuscript; Contributed data analysis; Wrote the paper.

### Funding statement

This research did not receive any specific grant from funding agencies in the public, commercial, or not-for-profit sectors.

### Data availability statement

Data will be made available on request.

### Declaration of interest’s statement

The authors declare no conflict of interest.

### Additional information

No additional information is available for this paper.
